# Ce-Modified MnCo_2_O_4_ Flower-like Nanosheet Electrodes via PVP-Assisted Assembly for MnCo_2_O_4_//Carbon-Supported Iron Oxide Asymmetric Supercapacitors

**DOI:** 10.3390/mi17070870

**Published:** 2026-07-22

**Authors:** Wei Xu, Changxu Qu, Mingzhao Xing, Tingting Hao, Jian Hao, Zheng Zhao, Jing Wang

**Affiliations:** 1 School of Light Industry, Harbin University of Commerce, Harbin 150028, China; 2 School of Chemical Engineering, Harbin Institute of Technology, Harbin 150001, China; 3 State Key Laboratory of High-Efficiency Utilization of Coal and Green Chemical Engineering, Ningxia University, Yinchuan 750021, China; 4 Beijing Institute of Nonferrous Metals Research, Beijing 100088, China

**Keywords:** MnCo_2_O_4_, Ce modification, PVP-assisted assembly, carbon-supported iron oxide, asymmetric supercapacitor, electrochemical energy storage

## Abstract

Ce-modified MnCo_2_O_4_ flower-like nanosheet electrodes were prepared on nickel foam by a hydrothermal-calcination route and sequentially optimized with respect to reaction time, nominal Ce content, and PVP addition. Comparative SEM, XRD, XPS, and N_2_-sorption analyses identify MnCo_2_O_4_-9 h-3%Ce-PVP as the optimized electrode, with an open hierarchical nanosheet network and a BET surface area of 210.0 m^2^ g^−1^. The direct XRD/XPS control comparison distinguishes Ce-associated lattice and surface-state changes from PVP-associated synthesis effects without treating either trend as proof of substitutional Ce occupancy or quantitatively established oxygen vacancies. Likewise, PVP is treated as a morphology-directing additive whose transient adsorption or bridging role remains a synthesis hypothesis rather than a directly verified molecular mechanism. The optimized positive electrode delivers 2008 F g^−1^ at 1 A g^−1^, retains 1227 F g^−1^ at 20 A g^−1^, and shows 99.0% capacitance retention after 10,000 cycles at 5 A g^−1^. A carbon-supported iron oxide negative electrode, designated C/Fe_2_O_3_ only as a sample label because its exact oxide phase was not independently resolved by XRD or Raman spectroscopy, provides 443 F g^−1^ at 1 A g^−1^. The resulting charge-balanced asymmetric device operates over 0–1.6 V and delivers 34.6 F g^−1^ at 1 A g^−1^, corresponding to 12.30 Wh kg^−1^ at 0.8 kW kg^−1^. At 10 A g^−1^, it retains 29.8 F g^−1^ and delivers 10.60 Wh kg^−1^ at 8.0 kW kg^−1^, equivalent to 86.1% capacitance retention over a tenfold increase in current density. All device-level gravimetric values are calculated using the total active mass of both electrodes.

## 1. Introduction

Supercapacitors are attractive electrochemical energy-storage devices because of their high power density, rapid charge–discharge response, long cycle life, and operational safety. Their lower energy density than secondary batteries nevertheless restricts applications that require simultaneous high energy and high power. Asymmetric supercapacitors address this limitation by pairing electrodes with complementary potential windows, thereby increasing the cell voltage and energy output while retaining fast electrochemical response [1,2,3,4].

Spinel transition-metal oxides, including NiCo_2_O_4_, ZnCo_2_O_4_, NiMn_2_O_4_, and MnCo_2_O_4_, are widely studied as pseudocapacitive electrodes because their multivalent cations support reversible Faradaic reactions. MnCo_2_O_4_ was selected here because it combines the redox activity of both Mn and Co with comparatively abundant, low-cost manganese and a robust spinel framework. Relative to highly conductive Ni-containing spinels, however, pristine MnCo_2_O_4_ commonly exhibits lower intrinsic electronic conductivity, incomplete exposure of active sites, and diffusion limitations at high current density. These limitations make MnCo_2_O_4_ a suitable platform for testing whether controlled lattice perturbation and hierarchical morphology regulation can improve accessible charge storage [5,6,7,8,9,10,11].

Moderate Ce introduction can perturb the MnCo_2_O_4_ lattice because Ce ions are substantially larger than the host transition-metal cations, while the Ce^3+^/Ce^4+^ couple can modify the local electronic and oxygen-coordination environment [12,13,14]. Such perturbation is potentially useful only within a limited composition range: excessive Ce can increase microstrain, disrupt coherent spinel growth, or promote poorly integrated Ce-rich domains. The nominal Ce content was therefore optimized rather than maximized. PVP is commonly used as a polymeric morphology regulator because its polar lactam groups can interact transiently with dissolved metal species and growing oxide precursors, thereby affecting nucleation, sheet restacking, and mesoscale assembly [15]. In the absence of direct adsorption measurements such as FTIR, TGA, or zeta-potential analysis, this molecular picture is retained only as a plausible synthesis pathway; the experimentally supported description is PVP-assisted morphology regulation. For the negative electrode, iron oxide supplies low-cost redox-active sites, whereas the carbon framework provides a continuous conductive pathway and can accommodate interfacial and volume changes during repeated polarization [16,17,18].

The specific gap addressed in this work is the absence of a controlled, sequential assessment of hydrothermal duration, moderate Ce modification, and PVP-assisted assembly within one MnCo_2_O_4_ electrode platform. Reaction time is optimized first, nominal Ce content second, and PVP addition third, while morphology, phase structure, surface chemical states, porosity, and electrochemical behavior are evaluated at the relevant stages. Within this sequence, PVP-assisted synthesis is evaluated specifically as a morphology-regulation step intended to reduce nanosheet restacking and increase accessible intersheet porosity, rather than as a proven molecular-bridging process. A MnCo_2_O_4_-9 h-PVP control is included in the direct XRD/XPS comparison so that Ce-associated and PVP-associated trends can be discussed separately rather than inferred from the optimized end-point sample alone. Ce-related changes are interpreted from comparative XRD and XPS without claiming quantitatively established oxygen vacancies or proven substitutional Ce occupancy. The optimized positive electrode is subsequently paired with a carbon-supported iron oxide negative electrode in a charge-balanced asymmetric device, and all device metrics are reported using the total active mass of both electrodes.

## 2. Materials and Methods

Flower-like MnCo_2_O_4_-based positive electrodes were prepared by hydrothermal synthesis followed by calcination. A controlled-variable strategy was used so that the effects of hydrothermal duration, nominal Ce content, and PVP addition could be evaluated sequentially rather than changed simultaneously.

Step 1: MnCo_2_O_4_ samples were synthesized for 6, 9, and 12 h to identify the hydrothermal duration that produced the most open and continuous nanosheet network.

Step 2: Using the optimized 9 h condition, Ce was introduced at nominal contents of 1, 3, and 5 mol% relative to the total Mn and Co amount to identify a moderate doping level.

Step 3: PVP was added to the optimized MnCo_2_O_4_-9 h-3%Ce precursor solution to evaluate its effect on nanosheet assembly and flower-like hierarchical organization. A Ce-free MnCo_2_O_4_-9 h-PVP control was prepared under the same PVP-assisted condition for comparative XRD and XPS analysis.

### 2.1. Preparation of MnCo_2_O_4_

First, 2 mmol of Mn(NO_3_)_2_·6H_2_O and 4 mmol of Co(NO_3_)_2_·6H_2_O were separately dissolved in deionized water and then combined to maintain a Mn:Co molar ratio of 1:2. Urea (15 mmol) was added as a slow OH^−^-release agent, and the solution was stirred for 30 min. Pretreated nickel foam (1 cm × 1 cm; active growth area, 1.0 cm^2^) was immersed in the precursor and transferred to a 100 mL Teflon-lined autoclave. Hydrothermal reactions were conducted at 180 °C for 6, 9, or 12 h. After natural cooling, the products were washed with deionized water and ethanol, dried at 60 °C for 8 h, and calcined at 350 °C for 2 h at 2 °C min^−1^. The product obtained after 9 h was denoted MnCo_2_O_4_-9 h.

### 2.2. Preparation of MnCo_2_O_4_-9 h-x%Ce

The Ce-containing samples were prepared by the same procedure, except that Ce(NO_3_)_3_·6H_2_O was added to the mixed precursor. Nominal Ce contents of 1, 3, and 5 mol% relative to the total Mn and Co amount were used, and the products were denoted MnCo_2_O_4_-9 h-1%Ce, MnCo_2_O_4_-9 h-3%Ce, and MnCo_2_O_4_-9 h-5%Ce, respectively. These percentages describe precursor ratios and should not be interpreted as quantitative bulk compositions determined by EDS.

### 2.3. Preparation of MnCo_2_O_4_-9 h-3%Ce-PVP

For the PVP-assisted sample, PVP K30 (111 mg, corresponding to approximately 1 mmol of N-vinylpyrrolidone repeat units; average molecular weight ≈ 40,000) was added to the MnCo_2_O_4_-9 h-3%Ce precursor before hydrothermal treatment. The hydrothermal, washing, drying, and calcination procedures were otherwise unchanged. The final product was denoted MnCo_2_O_4_-9 h-3%Ce-PVP. For the Ce-free control used in the direct XRD/XPS comparison, the same amount of PVP was added to the MnCo_2_O_4_-9 h precursor without Ce(NO_3_)_3_·6H_2_O, and the product was denoted MnCo_2_O_4_-9 h-PVP. Because no direct PVP adsorption measurement was performed, the term ‘PVP-assisted’ denotes the synthesis condition and observed assembly change rather than a proven molecular-bridging mechanism.

### 2.4. Preparation of the Carbon-Supported Iron Oxide Negative Electrode

**Figure 1:**
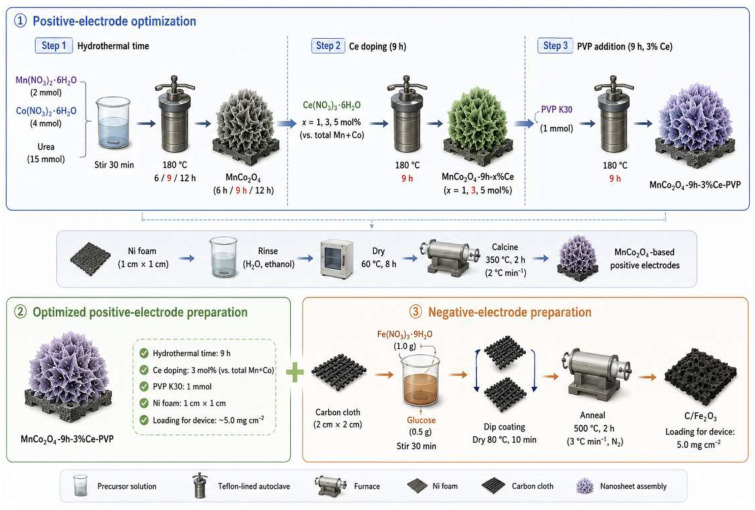
Overall design and fabrication scheme for the MnCo_2_O_4_-9 h-3%Ce-PVP//carbon-supported iron oxide asymmetric supercapacitor, including sequential optimization of the positive electrode, preparation of the optimized positive electrode, and fabrication of the carbon-supported iron oxide negative electrode. C/Fe_2_O_3_ is retained only as the negative-electrode sample label.

Carbon cloth was alternately rinsed with deionized water and ethanol, ultrasonicated for 15 min, and dried. Fe(NO_3_)_3_·9H_2_O (1.0 g) and glucose (0.5 g) were dissolved in 20 mL of deionized water and stirred for 30 min. A pretreated carbon-cloth substrate (2 cm × 2 cm) was repeatedly immersed in the precursor, removed, and dried at 80 °C for 10 min to build a uniform precursor layer. The coated substrate was annealed at 500 °C for 2 h under N_2_ at 3 °C min^−1^ to obtain a carbon-supported iron oxide electrode. The notation C/Fe_2_O_3_ is retained only as the original sample label associated with the precursor/annealing route; because phase-specific XRD or Raman data are not available for this electrode, the material is otherwise described as carbon-supported iron oxide. The active-material loading used for device assembly was 5.0 mg cm^−2^, as shown in Figure 1.

### 2.5. Assembly of the Supercapacitor

The asymmetric supercapacitor was assembled using MnCo_2_O_4_-9 h-3%Ce-PVP as the positive electrode and the carbon-supported iron oxide electrode (sample label: C/Fe_2_O_3_) as the negative electrode. Three-electrode measurements were conducted in 3 mol L^−1^ KOH using a Pt-foil counter electrode and an Hg/HgO reference electrode. Positive-electrode CV measurements were performed over 0–0.60 V. This upper limit was adopted as a conservative comparison window so that high-potential current potentially influenced by parasitic oxidation was not included in the pseudocapacitive analysis; it is not presented as the absolute stability limit of the electrode. GCD calculations used the effective 0–0.50 V discharge interval after excluding the initial IR drop. The negative electrode was evaluated over −1.0–0 V; for charge balancing, its effective GCD discharge interval after the initial voltage drop was approximately 0.91 V. The two-electrode device was evaluated over 0–1.6 V. At 1 A g^−1^, Equation (3), using C_+_ = 2008 F g^−1^, C_−_ = 443 F g^−1^, and the effective discharge intervals, gave a target positive/negative active-mass ratio of approximately 0.40. Practical loadings of 2.1 and 5.0 mg cm^−2^ gave a ratio of 0.42, within approximately 5% of the calculated target, and a total active mass of 7.1 mg for a 1 cm^2^ device. Nickel foam and carbon cloth were excluded from the gravimetric calculations.

q = C_s_ × m × ΔV (1)

C_s_ = I × Δt/(m × ΔV) (2)

m^+^/m^−^ = (C_−_ × ΔV_−_)/(C_+_ × ΔV_+_) (3)

E = C_device_ × (ΔV)^2^/(2 × 3.6) (4)

P = E × 3600/Δt(5)

Charge balance was determined from q = C_s_mΔV. Electrode capacitances were calculated from the discharge branch after excluding the initial IR drop. Device current densities, capacitance, energy density, and power density were normalized to the combined active mass of the positive and negative electrodes. The reported gravimetric values therefore exclude current collectors, separator, electrolyte, and packaging, and should be interpreted as electrode-level rather than packaged-cell metrics.

### 2.6. Characterization and Data-Interpretation Criteria

Morphology and local elemental distribution were evaluated by SEM, TEM, HRTEM, SAED, and EDS; crystalline structure was assessed by XRD; surface chemical states were compared by XPS; and pore properties were assessed by N_2_ adsorption–desorption analysis. EDS mapping and spectra are treated as local, qualitative compositional evidence and are not used to determine bulk Ce content. Comparative XPS survey and high-resolution spectra were obtained for MnCo_2_O_4_-9 h, MnCo_2_O_4_-9 h-3%Ce, MnCo_2_O_4_-9 h-PVP, and MnCo_2_O_4_-9 h-3%Ce-PVP; Ce 3d spectra were compared for the two Ce-containing samples, and detailed peak fitting is presented only for the optimized MnCo_2_O_4_-9 h-3%Ce-PVP electrode. Cross-sample changes in peak position and envelope shape are used as qualitative evidence of different surface chemical environments, whereas fitted O 1s components are not converted into oxygen-vacancy concentrations and fitted valence-state areas are not used as absolute stoichiometric fractions. EIS equivalent circuits are used to compare apparent interfacial responses rather than to establish intrinsic bulk conductivity. For all GCD calculations, only the relevant discharge segment is used. These criteria prevent morphological, spectroscopic, and electrochemical trends from being interpreted beyond the resolution of the available measurements.

## 3. Results and Discussion

### 3.1. Morphology Evolution and PVP-Assisted Hierarchical Assembly of MnCo_2_O_4_-Based Electrodes

**Figure 2:**
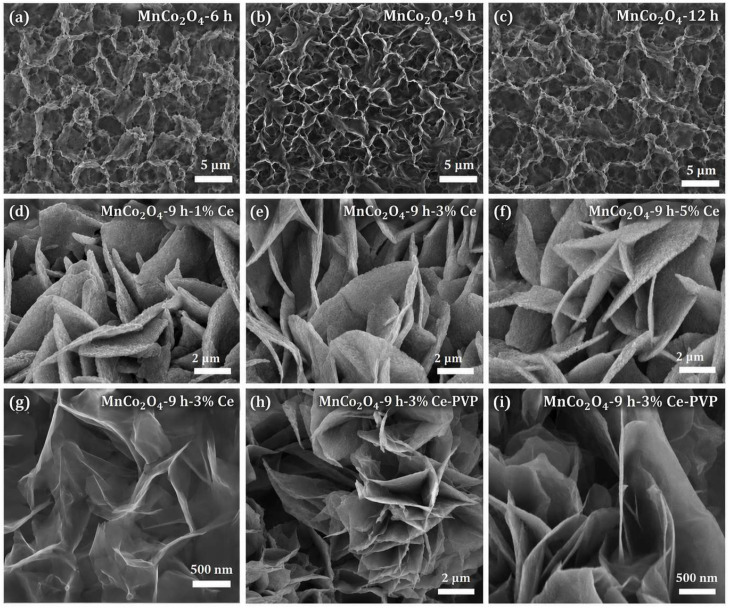
SEM images illustrating the sequential optimization of MnCo_2_O_4_-based electrodes: (**a**) MnCo_2_O_4_-6 h, (**b**) MnCo_2_O_4_-9 h, (**c**) MnCo_2_O_4_-12 h, (**d**) MnCo_2_O_4_-9 h-1%Ce, (**e**) MnCo_2_O_4_-9 h-3%Ce, (**f**) MnCo_2_O_4_-9 h-5%Ce, (**g**) higher-magnification MnCo_2_O_4_-9 h-3%Ce, and (**h**,**i**) MnCo_2_O_4_-9 h-3%Ce-PVP at low and high magnification.

Figure 2 summarizes the complete SEM-based optimization sequence. The time-controlled samples are compared in Figure 2a–c, the nominal Ce-content series in Figure 2d–f, and the samples before and after PVP addition in Figure 2g–i.

After 6 h, the oxide network remains comparatively irregular and locally aggregated (Figure 2a). Extending the reaction to 9 h produces a more continuous open nanosheet network with interconnected voids (Figure 2b), whereas 12 h leads to denser regions and partial restacking (Figure 2c). Within the Ce series, 1% Ce gives relatively loosely organized sheets, 3% Ce yields the most uniform and separated nanosheets, and 5% Ce produces thicker or more closely stacked features (Figure 2d–f). The non-monotonic morphology supports an optimum-modification interpretation: moderate Ce-associated perturbation is compatible with open sheet growth, whereas the highest nominal content increases structural disorder and aggregation. The higher-magnification image confirms the thin, wrinkled character of the 3% Ce nanosheets (Figure 2g). PVP addition changes the assembly toward more curled, flower-like aggregates with open intersheet spaces (Figure 2h,i). Such an architecture can increase electrolyte-accessible surface and provide multiple ion-entry pathways without requiring the sheets themselves to be intrinsically more conductive. SEM alone cannot establish molecular adsorption or bridging; therefore, the images support a PVP-associated assembly change but not a specific molecular interaction geometry.

**Figure 3:**
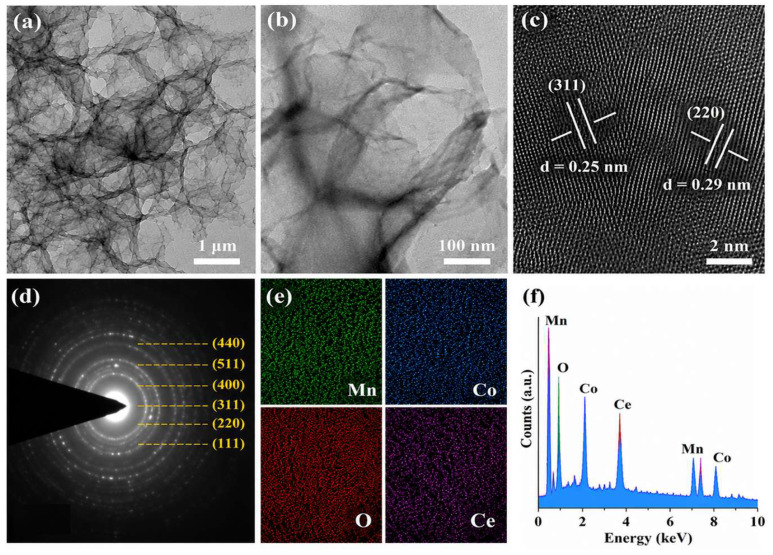
TEM characterization of MnCo_2_O_4_-9 h-3%Ce-PVP: (**a**,**b**) TEM images; (**c**) HRTEM image with measured lattice fringes; (**d**) SAED pattern; (**e**) elemental maps of Mn, Co, O, and Ce; and (**f**) EDS spectrum.

TEM confirms that the optimized material retains an ultrathin, interconnected nanosheet framework (Figure 3a,b). The HRTEM fringes of approximately 0.25 and 0.29 nm are consistent with the (311) and (220) planes, respectively, of spinel MnCo_2_O_4_ (Figure 3c), in agreement with reported MnCo_2_O_4_ lattice spacings [19,20]. The indexed SAED rings indicate a polycrystalline spinel structure (Figure 3d). Mn, Co, O, and Ce are spatially distributed throughout the mapped region (Figure 3e). The relatively intense Ce signal in the local EDS spectrum (Figure 3f) should not be used to infer bulk Ce content because EDS intensity is affected by sampling position, specimen thickness, detector geometry, and the selected analysis region. Accordingly, the nominal 3 mol% value refers to the precursor composition, and the EDS result is used only as qualitative evidence that Ce is present in the analyzed nanosheet region.

### 3.2. Morphology and Microstructure of the Carbon-Supported Iron Oxide Negative Electrode

**Figure 4:**
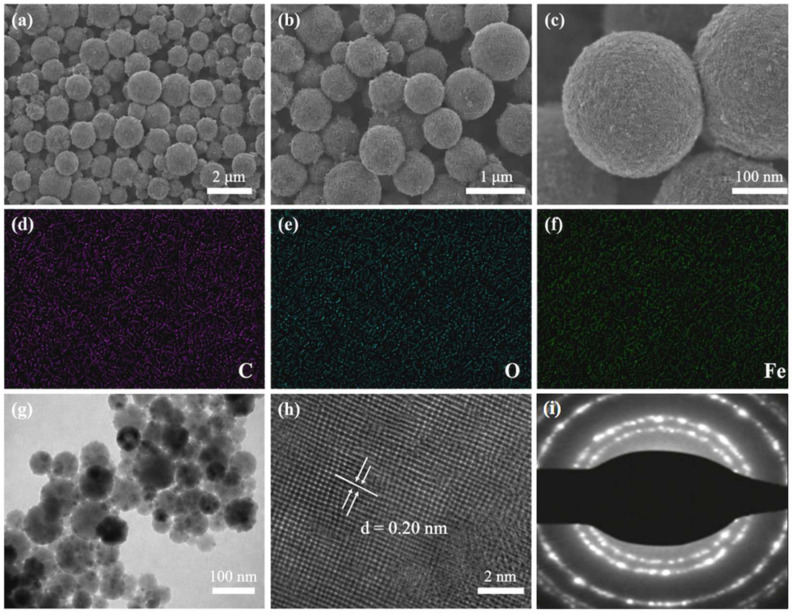
Morphology and microstructure of the carbon-supported iron oxide electrode (sample label: C/Fe_2_O_3_): (**a**–**c**) SEM images; (**d**–**f**) C, O, and Fe elemental maps; (**g**) TEM image; (**h**) HRTEM image showing an approximately 0.20 nm lattice spacing; and (**i**) SAED pattern.

The carbon-supported negative electrode consists of densely distributed, rough-surfaced iron-containing oxide particles (Figure 4a–c). Elemental maps show C, O, and Fe throughout the selected region (Figure 4d–f), consistent with oxide particles integrated with a carbon framework. TEM reveals aggregated nanoscale particles (Figure 4g). The approximately 0.20 nm lattice spacing and ring-like SAED pattern confirm crystalline domains (Figure 4h,i), but they do not uniquely identify an iron-oxide polymorph. Recent interfacial-materials studies illustrate the broader importance of combining local lattice imaging with complementary structural and interface-sensitive evidence rather than relying on a single fringe [21]. Because the material system in that study is not an iron oxide, its lattice spacing is not used as a phase-specific standard here. A directly relevant iron-oxide study reports a 0.210 nm spacing for the (400) planes of mixed Fe_3_O_4_-γ-Fe_2_O_3_ nanoparticles [22]. Accordingly, and because no phase-specific XRD or Raman pattern is available for the present negative electrode, Figure 4 is not used to claim an unambiguous Fe_2_O_3_ phase; the material is conservatively termed carbon-supported iron oxide.

### 3.3. Crystal Structure and Comparative Surface Chemical States of MnCo_2_O_4_-Based Electrodes

**Figure 5:**
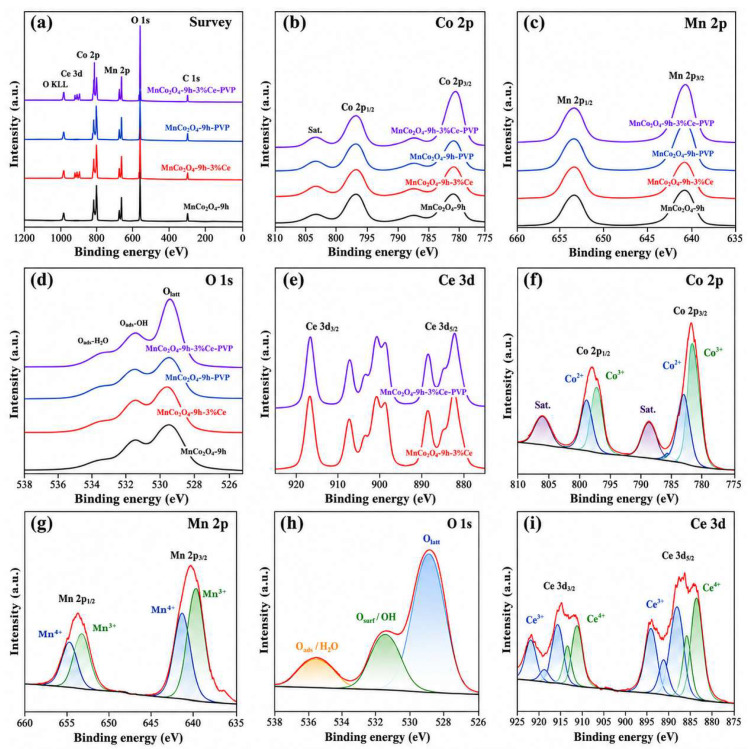
Comparative XPS spectra of MnCo_2_O_4_-based electrodes: (**a**) survey, (**b**) Co 2p, (**c**) Mn 2p, and (**d**) O 1s spectra of MnCo_2_O_4_-9 h, MnCo_2_O_4_-9 h-3%Ce, MnCo_2_O_4_-9 h-PVP, and MnCo_2_O_4_-9 h-3%Ce-PVP; (**e**) Ce 3d spectra of the two Ce-containing samples; and fitted (**f**) Co 2p, (**g**) Mn 2p, (**h**) O 1s, and (**i**) Ce 3d spectra of the optimized MnCo_2_O_4_-9 h-3%Ce-PVP electrode. The comparative spectra are vertically offset for clarity. Fitted components are used for qualitative surface-state assignments and not for absolute stoichiometric, Ce-valence-fraction, or oxygen-vacancy quantification.

The comparative survey spectra contain Mn, Co, and O signals for all four electrodes, while the Ce 3d signal appears only in the two Ce-containing samples (Figure 5a). This control comparison confirms that the detected Ce signal is associated with Ce precursor addition rather than with the PVP-assisted route. The Co 2p and Mn 2p envelopes exhibit modest changes in peak position and line shape after Ce introduction and/or PVP-assisted synthesis (Figure 5b,c). For the optimized electrode, the fitted Co 2p spectrum assigns the lower-binding-energy components to Co^3+^ and the slightly higher-binding-energy components to Co^2+^, together with shake-up satellites (Figure 5f), consistent with Co-containing spinel oxides [23,24]. In the fitted Mn 2p spectrum, the higher-binding-energy components are assigned to Mn^4+^ and the lower-binding-energy components to Mn^3+^ (Figure 5g), in agreement with reported MnCo_2_O_4_ analyses [24]. These component assignments are specific to the fitted spinel environment. More generally, core-level positions depend on local coordination, metal–ligand charge redistribution, energy referencing, and final-state screening; therefore, the observed intersample shifts are interpreted as qualitative surface–electronic modulation rather than as a direct measure of charge transfer or bulk oxidation-state fractions [25,26]. The comparative O 1s spectra show that the relative lattice-oxygen and higher-binding-energy surface contributions vary among the four samples (Figure 5d). In the optimized-sample fit, these components are described as lattice oxygen, surface oxygen/hydroxyl species, and adsorbed H_2_O-related species (Figure 5h). Because the components overlap and neither EPR nor a calibrated vacancy standard was used, the higher-binding-energy contribution is not equated with, or converted into, an oxygen-vacancy concentration. The Ce 3d envelopes of MnCo_2_O_4_-9 h-3%Ce and MnCo_2_O_4_-9 h-3%Ce-PVP both contain Ce^3+^- and Ce^4+^-associated features, with modest line-shape differences after PVP-assisted synthesis (Figure 5e). The detailed fit of the optimized sample likewise supports mixed Ce^3+^/Ce^4+^ surface states (Figure 5i), but the fitted areas are not treated as absolute Ce valence fractions. Thus, the updated four-sample comparison permits Ce-associated and PVP-associated surface trends to be distinguished more directly, while still avoiding claims of quantitative vacancy generation, proven substitutional Ce occupancy, or a specific PVP adsorption geometry.

**Figure 6:**
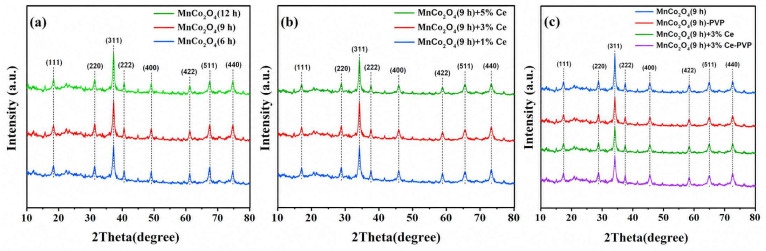
XRD patterns of MnCo_2_O_4_-based samples: (**a**) MnCo_2_O_4_ prepared for 6, 9, and 12 h; (**b**) MnCo_2_O_4_-9 h containing nominally 1, 3, and 5% Ce; and (**c**) direct comparison of MnCo_2_O_4_-9 h, MnCo_2_O_4_-9 h-PVP, MnCo_2_O_4_-9 h-3%Ce, and MnCo_2_O_4_-9 h-3%Ce-PVP. The spinel reflections at approximately 18.8°, 31.0°, 36.3°, 38.0°, 44.1°, 54.8°, 58.4°, and 64.3° are assigned to the (111), (220), (311), (222), (400), (422), (511), and (440) planes, respectively. Intense features near 44.5°, 51.8°, and 76.4° contain major contributions from the nickel-foam substrate; the spinel (400) reflection overlaps the substrate contribution near 44–45°.

All three XRD panels retain the characteristic cubic-spinel reflection sequence reported for MnCo_2_O_4_ [5,10,19,20]. In the time series, the 9 h sample shows more distinct oxide reflections than the 6 h material, whereas extending the treatment to 12 h provides no clear structural advantage (Figure 6a). The 1–5% Ce series preserves the same spinel framework without a clearly resolved crystalline CeO_2_ impurity peak (Figure 6b). Small changes in peak position and breadth are compatible with local distortion or microstrain associated with the Ce-containing synthesis, but are not proof of substitutional Ce occupancy. The strongest substrate-dominated features near 44.5°, 51.8°, and 76.4° are not used to infer changes in MnCo_2_O_4_ crystallinity. The four-sample control comparison in Figure 6c shows that MnCo_2_O_4_-9 h-PVP remains close to the undoped MnCo_2_O_4_-9 h pattern and introduces no resolvable new crystalline phase. The two Ce-containing samples also retain the spinel framework, while their modest peak-position and breadth differences are consistent with Ce-associated lattice perturbation within the resolution of the measurement. No additional crystalline phase appears in MnCo_2_O_4_-9 h-3%Ce-PVP relative to MnCo_2_O_4_-9 h-3%Ce. Accordingly, the direct comparison supports assigning the principal PVP effect to morphology/assembly and accessible porosity, whereas the structural perturbation is associated mainly with the Ce-containing synthesis. It does not, however, establish substitutional Ce occupancy, a precise lattice parameter change, or a crystalline-phase transformation.

### 3.4. Porosity and Electrochemical Performance of the Positive Electrode

**Figure 7:**
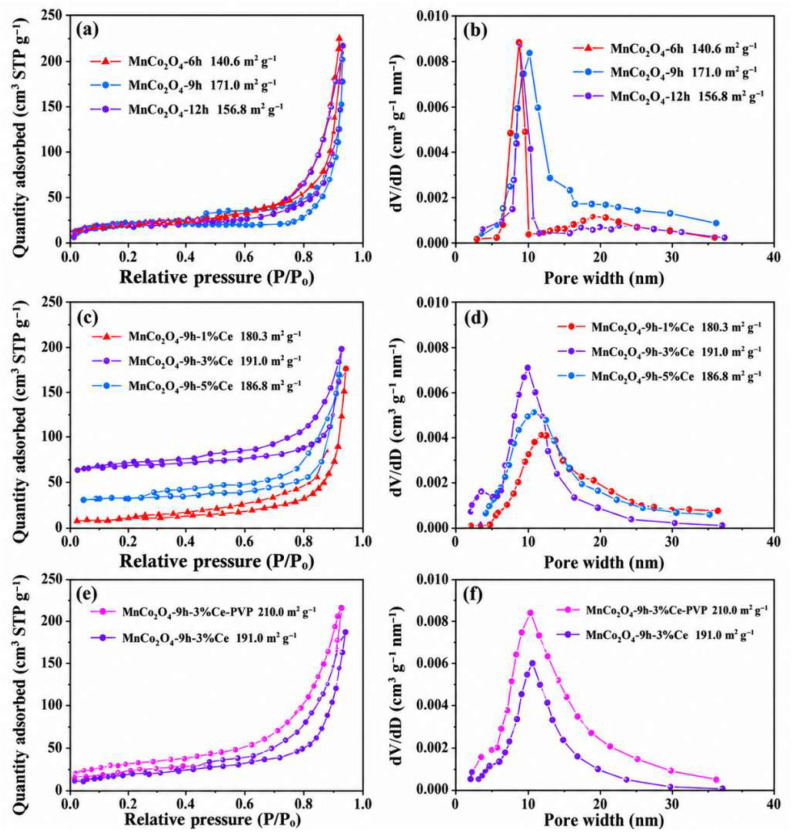
N_2_ adsorption–desorption analysis of MnCo_2_O_4_-based samples: (**a**,**c**,**e**) adsorption–desorption isotherms and (**b**,**d**,**f**) pore-size distributions for the reaction-time, Ce-content, and PVP-assisted comparisons, respectively.

Nitrogen adsorption–desorption measurements further distinguish the three optimization stages (Figure 7). The BET surface areas are 140.6, 171.0, and 156.8 m^2^ g^−1^ for MnCo_2_O_4_-6 h, MnCo_2_O_4_-9 h, and MnCo_2_O_4_-12 h, respectively (Figure 7a), confirming that 9 h provides the most accessible surface among the time-controlled samples. Their pore-size distributions are centered mainly in the mesoporous range, with the 9 h material showing the strongest accessible pore contribution (Figure 7b). For the Ce series, the areas increase to 180.3, 191.0, and 186.8 m^2^ g^−1^ for 1, 3, and 5% Ce, respectively (Figure 7c), while the corresponding distributions confirm that moderate 3% Ce gives the most favorable mesopore population (Figure 7d). PVP-assisted assembly further raises the area from 191.0 to 210.0 m^2^ g^−1^ and increases the accessible mesoporous contribution (Figure 7e,f). These trends support improved electrolyte contact and shorter diffusion paths, although BET data alone do not establish electronic conductivity.

The combined SEM and sorption results therefore indicate that the performance improvement is associated with a balance between exposed nanosheet surface, intersheet mesoporosity, and avoidance of excessive restacking. This structural interpretation is used below together with electrochemical kinetics; it is not treated as direct evidence of a PVP molecular-bridging mechanism.

**Figure 8:**
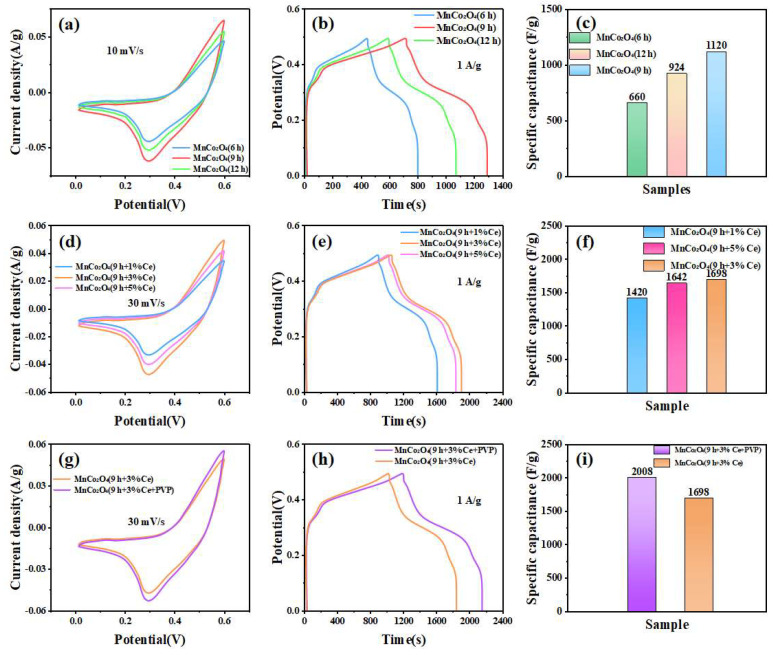
Sequential three-electrode electrochemical comparison of MnCo_2_O_4_-based positive electrodes: (**a**–**c**) reaction-time series, (**d**–**f**) nominal Ce-content series, and (**g**–**i**) MnCo_2_O_4_-9 h-3%Ce before and after PVP-assisted synthesis. Panels (**a**,**d**,**g**) show CV curves, (**b**,**e**,**h**) show GCD curves at 1 A g^−1^, and (**c**,**f**,**i**) show the corresponding specific capacitances.

Figure 8 compares the electrochemical response at each optimization stage. The CV curves were recorded over the conservative 0–0.60 V comparison window, whereas the GCD-derived capacitances were calculated from the effective 0–0.50 V discharge interval at 1 A g^−1^ after exclusion of the initial IR drop. For the reaction-time series, MnCo_2_O_4_-9 h shows the largest CV area and longest discharge segment, giving 1120 F g^−1^ compared with 660 and 924 F g^−1^ for 6 and 12 h, respectively (Figure 8a–c). For the Ce series, the 3% sample provides the strongest response, with 1698 F g^−1^ compared with 1420 and 1642 F g^−1^ for 1 and 5% Ce (Figure 8d–f). PVP-assisted synthesis further increases the capacitance from 1698 to 2008 F g^−1^ (Figure 8g–i). Each value is calculated from the corresponding discharge branch after exclusion of the initial IR drop, ensuring direct consistency between the GCD and capacitance panels.

C_s_ = I × Δt/(m × ΔV) (6)

The sequential trend is consistent across CV, GCD, and calculated capacitance: 9 h provides a more accessible nanosheet network, moderate 3% Ce gives a favorable balance between lattice perturbation and structural integrity, and PVP-assisted synthesis increases the open hierarchical organization. These observations support improved accessible charge storage, but they do not constitute a direct measurement of intrinsic electronic conductivity.

**Figure 9:**
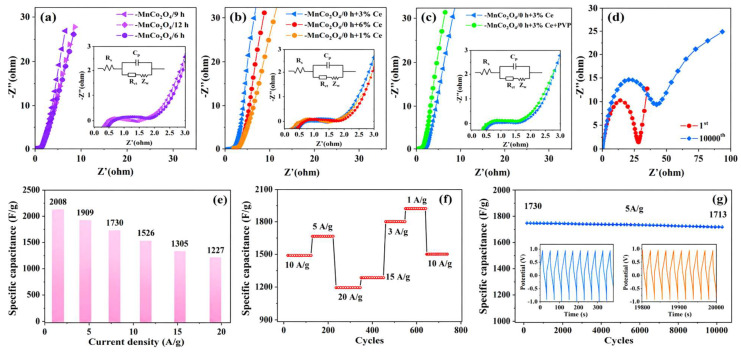
Electrochemical kinetics, rate behavior, and cycling stability of MnCo_2_O_4_-based positive electrodes: Nyquist plots for (**a**) the reaction-time series, (**b**) the Ce-content series, and (**c**) MnCo_2_O_4_-9 h-3%Ce before and after PVP addition; (**d**) Nyquist plots of the optimized electrode before and after 10,000 cycles; (**e**) specific capacitance versus current density; (**f**) rate-recovery test; and (**g**) cycling stability at 5 A g^−1^, with initial and final GCD profiles shown as insets.

The Nyquist comparisons show that the 9 h, 3% Ce, and PVP-assisted samples have smaller apparent high-frequency semicircles and steeper low-frequency responses than their respective controls (Figure 9a–c), indicating lower interfacial charge-transfer resistance and more favorable apparent ion-transport kinetics. The equivalent circuit is used as a qualitative fitting model; because no independent four-probe conductivity measurement was performed, these EIS trends are not described as direct proof of increased intrinsic material conductivity. The optimized electrode delivers 2008, 1909, 1730, 1526, 1305, and 1227 F g^−1^ at 1, 3, 5, 10, 15, and 20 A g^−1^, respectively (Figure 9e), and the rate-recovery sequence returns close to the initial 10 A g^−1^ value after high-current cycling (Figure 9f). At 5 A g^−1^, the capacitance decreases from 1730 to 1713 F g^−1^ after 10,000 cycles, corresponding to 99.0% retention (Figure 9g). The post-cycling Nyquist plot shows a measurable increase in impedance (Figure 9d), indicating interfacial aging even though the capacitance retention remains high. Because post-cycling SEM or XRD was not obtained, structural preservation is not claimed directly from the electrochemical retention alone.

**Figure 10:**
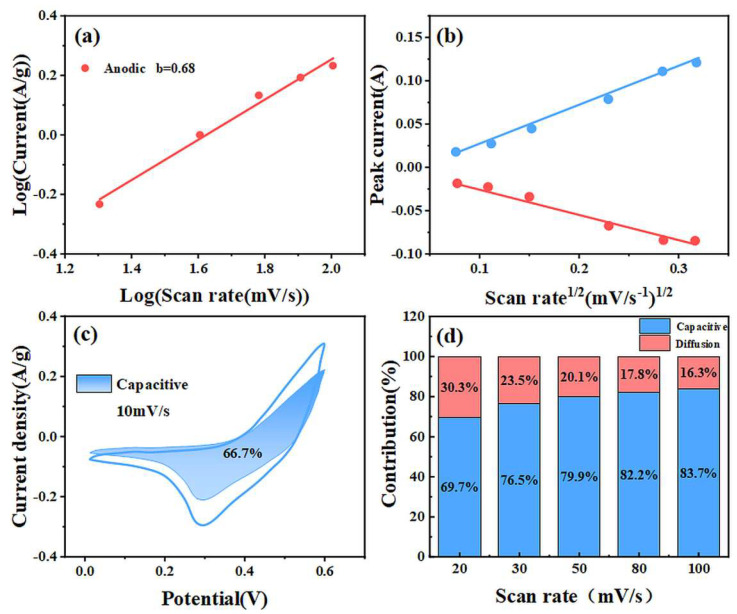
Kinetic analysis of MnCo_2_O_4_-9 h-3%Ce-PVP: (**a**) anodic-peak log(i)-log(v) fitting; (**b**) anodic- and cathodic-peak current density versus v^1^ᐟ^2^; (**c**) capacitive-controlled contribution at 10 mV s^−1^; and (**d**) capacitive-controlled and diffusion-controlled contribution ratios at different scan rates.

The charge-storage kinetics were evaluated using the power-law relationship i = av^b^ and the Dunn separation i(V) = k_1_v + k_2_v^1^ᐟ^2^, following established analyses of mixed diffusion-controlled and surface-controlled electrochemical storage [27,28]. The b-value analysis in Figure 10a uses the anodic peak as a representative feature; the slope is 0.68, between the ideal diffusion-controlled value of 0.5 and surface-controlled value of 1.0.

i = aν^b^ (7)

log(i) = b log(ν) + log(a)(8)

The fitted b value therefore indicates mixed charge storage involving both diffusion-influenced Faradaic reactions and surface or near-surface capacitive processes. The approximately linear peak-current-density relationships with v^1^ᐟ^2^ in Figure 10b are consistent with a substantial diffusion contribution. However, an absolute diffusion coefficient is not calculated because the electron-transfer number, electrochemically active area, and effective reactant concentration were not independently established; the v^1^ᐟ^2^ analysis is used only as comparative kinetic evidence.

At 10 mV s^−1^, the integrated capacitive-controlled fraction is 66.7% (Figure 10c). It increases from 69.7% at 20 mV s^−1^ to 83.7% at 100 mV s^−1^ (Figure 10d), while the diffusion-controlled fraction decreases correspondingly. The higher surface-controlled contribution at faster scans is consistent with rapid reactions at the exposed nanosheet surfaces and near-surface regions, whereas deeper ion access contributes more strongly at lower scan rates.

The kinetic analysis supports the rate performance of the optimized electrode, but the observed improvement is attributed to apparent electrochemical transport and accessible reaction sites rather than a directly measured change in intrinsic electrical conductivity.

i(V) = k_1_ν + k_2_ν^1/2^ (9)

Taken together, the data indicate a mixed mechanism in which the open nanosheet hierarchy improves electrolyte infiltration and redox-site accessibility, moderate Ce incorporation perturbs the local surface/electronic environment, and the PVP-assisted route changes nanosheet assembly. These contributions are experimentally supported at the morphology, XRD, XPS, porosity, EIS, and kinetic levels, while oxygen-vacancy generation and a specific PVP adsorption geometry remain unquantified.

MnCo_2_O_4_-9 h-3%Ce-PVP was therefore selected as the positive electrode for asymmetric-device construction with the carbon-supported iron oxide negative electrode.

### 3.5. Structural and Electrochemical Properties of the Carbon-Supported Iron Oxide Negative Electrode

The negative electrode was included to provide a complementary potential window and charge balance for the optimized MnCo_2_O_4_ positive electrode. Its discussion is therefore focused on the experimentally supported comparison with the Fe oxide control, while the exact iron-oxide phase remains conservatively unresolved.

**Figure 11:**
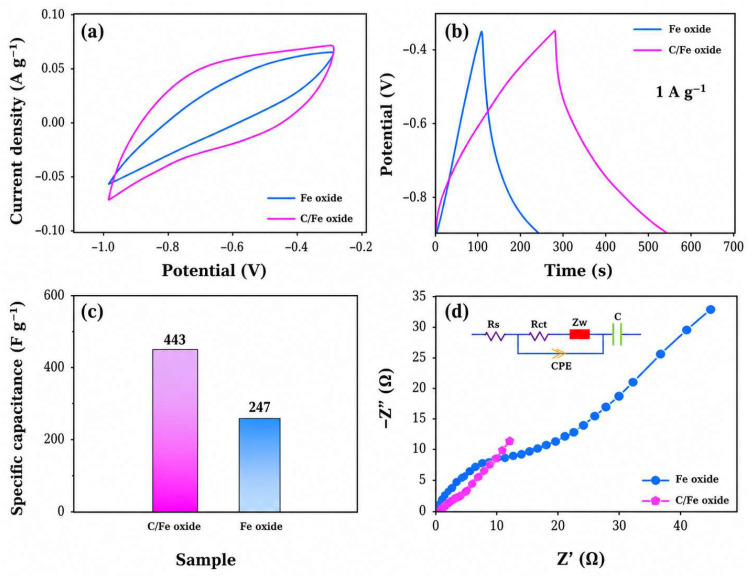
Electrochemical comparison of the carbon-supported iron oxide electrode (sample label: C/Fe_2_O_3_) and the Fe oxide control in a three-electrode system: (**a**) CV curves; (**b**) GCD curves at 1 A g^−1^; (**c**) specific capacitance at 1 A g^−1^; and (**d**) Nyquist plots with the equivalent circuit used for qualitative fitting.

The carbon-supported iron oxide electrode has a larger CV area and longer discharge segment than the Fe oxide control (Figure 11a,b). At 1 A g^−1^, the corresponding capacitances are 443 and 247 F g^−1^, respectively (Figure 11c). The carbon-supported sample also shows a smaller apparent high-frequency semicircle and a steeper low-frequency branch (Figure 11d), consistent with lower interfacial resistance and improved apparent ion/electron transport through the composite electrode. Because no stand-alone conductivity measurement was performed, this difference is attributed to the composite-level EIS response rather than quantified intrinsic conductivity of the oxide phase.

**Figure 12:**
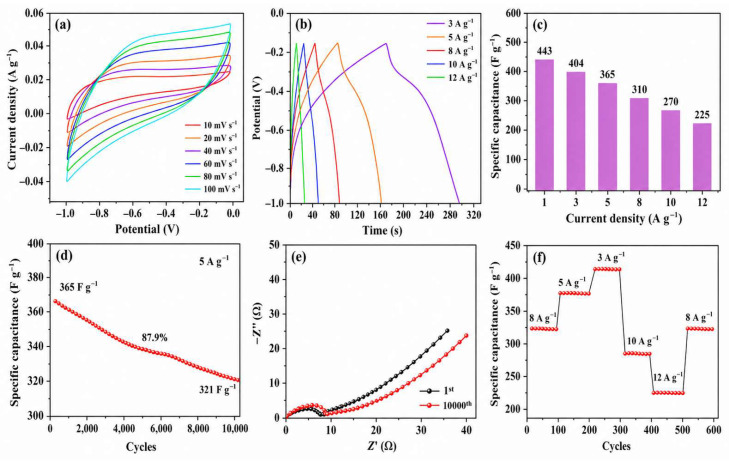
Electrochemical behavior of the carbon-supported iron oxide electrode (sample label: C/Fe_2_O_3_): (**a**) CV curves at different scan rates; (**b**) GCD profiles at different current densities; (**c**) specific capacitance; (**d**) cycling durability at 5 A g^−1^; (**e**) EIS spectra before and after cycling; and (**f**) rate recovery.

The negative electrode retains its redox-type CV response as the scan rate increases (Figure 12a). The GCD curves and calculated capacitances are internally consistent, giving 443, 404, 365, 310, 270, and 225 F g^−1^ at 1, 3, 5, 8, 10, and 12 A g^−1^, respectively (Figure 12b,c). At 5 A g^−1^, the capacitance decreases from 365 to 321 F g^−1^ after 10,000 cycles, corresponding to 87.9% retention (Figure 12d). The post-cycling EIS response changes only moderately and may include electrode activation and interfacial rearrangement (Figure 12e); without post-cycling structural characterization, it is not used as proof that a specific iron-oxide phase or morphology is fully preserved. The recovery to approximately 310 F g^−1^ when the current density returns to 8 A g^−1^ confirms reversible rate behavior (Figure 12f).

### 3.6. Device-Level Performance of the MnCo_2_O_4_-9 h-3%Ce-PVP//Carbon-Supported Iron Oxide Asymmetric Supercapacitor

The asymmetric device was assembled using a practical positive/negative active-mass ratio of 0.42, close to the charge-balance target of approximately 0.40. All device current-density, capacitance, energy-density, and power-density values use the combined 7.1 mg active mass of both electrodes. Current collectors, separator, electrolyte, and packaging are excluded, so the values represent an electrode-level mass basis rather than a packaged-cell basis.

**Figure 13:**
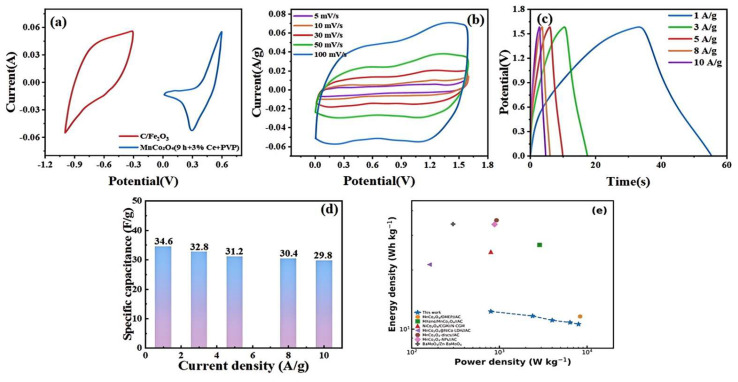
Electrochemical performance of the MnCo_2_O_4_-9 h-3%Ce-PVP//carbon-supported iron oxide asymmetric supercapacitor: (**a**) complementary operating windows of the positive and negative electrodes; (**b**) device CV curves at different scan rates over 0–1.6 V; (**c**) GCD curves at different current densities; (**d**) device specific capacitance calculated using the total active mass of both electrodes; and (**e**) Ragone comparison with recent MnCo_2_O_4_- and oxide-based asymmetric devices [7,10,29,30,31,32,33]. The five ‘This work’ points in (**e**) are calculated directly from the five GCD-derived capacitances in (**d**).

The complementary positive- and negative-electrode responses are shown in Figure 13a and support a 1.6 V device window. The device CV curves retain their broad redox features as the scan rate increases (Figure 13b), indicating reversible mixed Faradaic and capacitive charge storage rather than an ideal electric-double-layer response. The progressive current increase and modest shape distortion at a high scan rate reflect polarization and finite ion-transport time, while the absence of an abrupt current upturn within 0-1.6 V supports use of this window for quantitative analysis.

The nonlinear GCD profiles are consistent with the redox-type CV response and remain reproducible from 1 to 10 A g^−1^ (Figure 13c). Using only the discharge branch over the 1.6 V operating window, the device delivers 34.6, 32.8, 31.2, 30.4, and 29.8 F g^−1^ at 1, 3, 5, 8, and 10 A g^−1^, respectively (Figure 13d). The device therefore retains 86.1% of its 1 A g^−1^ capacitance when the current density is increased tenfold. This relatively small loss indicates that the complementary electrode pair maintains accessible charge storage under high-rate operation, even though the absolute device capacitance is lower than that of the positive electrode measured in a three-electrode configuration.

Using ΔV = 1.6 V and the total-active-mass capacitance, the corresponding energy densities are 12.30, 11.66, 11.09, 10.81, and 10.60 Wh kg^−1^ at power densities of 0.8, 2.4, 4.0, 6.4, and 8.0 kW kg^−1^, respectively (Figure 13e). Thus, 86.1% of the initial energy density is retained when the power density increases by one order of magnitude. The main device-level advantage is therefore a comparatively flat energy–power response and useful high-power energy retention, rather than a record maximum gravimetric energy density.

The literature points in Figure 13e were selected from recent MnCo_2_O_4_-containing, spinel-oxide, and closely related aqueous asymmetric systems [7,10,29,30,31,32,33]. Several composite systems report higher maximum energy densities, including MXene/MnCo_2_O_4_//AC, MnCo_2_O_4_@NiCo-LDH//AC, disc-like MnCo_2_O_4_//AC, and porous MnCo_2_O_4_ nanoplatelet//AC devices [10,29,31,32]. In contrast, the MnCo_2_O_4_/OMEP//AC device reports 11.6 Wh kg^−1^ at 8.33 kW kg^−1^ [7], close to the present value of 10.60 Wh kg^−1^ at 8.0 kW kg^−1^. The BaMoO_4_/Zn-BaMoO_4_ study suggested for comparison is also included [33]; its plotted Ragone point is calculated from the reported 76 F g^−1^, 1.8 V, and 0.33 A g^−1^ because that article emphasizes capacitance and cycling rather than directly tabulating energy and power density.

These comparisons must be interpreted cautiously because voltage window, electrolyte, electrode loading, current-density definition, and mass-normalization convention differ among reports. The present calculation uses the total active mass of both electrodes and a conservative 1.6 V aqueous window. Accordingly, the device is not claimed to outperform every reported oxide-based asymmetric supercapacitor. Its experimentally supported strengths are the high capacitance and cycling durability of the optimized positive electrode, 86.1% device-capacitance retention from 1 to 10 A g^−1^, and retention of 10.60 Wh kg^−1^ at 8.0 kW kg^−1^ on a transparent total-active-mass basis.

Table 1 compares the present results with the same recent devices plotted in Figure 13e, with emphasis on device voltage, energy/power output, cycling, and normalization basis. The comparison intentionally includes both higher-energy composite systems and the high-power MnCo_2_O_4_/OMEP benchmark so that the advantages and limitations of the present device are stated without selective performance claims.

The comparison shows that the present device occupies a high-power, moderate-energy region. Its maximum energy density is lower than that of several MnCo_2_O_4_ composite devices, but the decrease from 12.30 Wh kg^−1^ at 0.8 kW kg^−1^ to 10.60 Wh kg^−1^ at 8.0 kW kg^−1^ is limited to 13.9%. Combined with the positive electrode’s 2008 F g^−1^ capacitance and 99.0% retention after 10,000 cycles, this result supports the value of the sequential synthesis strategy for obtaining a highly active and rate-capable positive electrode while maintaining useful device output. The carbon-supported negative electrode, explicit total-mass normalization, and conservative phase and defect interpretation further improve the transparency of the comparison.

Several limitations remain and are stated explicitly. No EPR measurement was performed to quantify oxygen vacancies; PVP adsorption was not independently verified by FTIR, TGA, or zeta-potential analysis; the negative-electrode phase was not resolved by XRD or Raman spectroscopy; intrinsic electronic conductivity was not measured separately; and post-cycling SEM or XRD was not obtained. Consequently, the manuscript does not claim quantified vacancy formation, a unique molecular PVP-bridging geometry, a single Fe_2_O_3_ polymorph, an intrinsic-conductivity enhancement, or direct post-cycling preservation of morphology. The electrochemical retention data establish operational durability, whereas structural durability remains to be verified in future work. Device metrics also exclude inactive cell components, and future studies should address packaged-cell behavior, practical areal loading, temperature tolerance, and full-device long-term cycling.

## 4. Conclusions

A sequential optimization strategy was used to construct a Ce-modified MnCo_2_O_4_ flower-like nanosheet electrode. A 9 h hydrothermal treatment produced the most accessible time-controlled nanosheet network, nominal 3% Ce gave the most favorable balance of structural perturbation and electrochemical response, and PVP-assisted synthesis increased hierarchical assembly and BET surface area from 191.0 to 210.0 m^2^ g^−1^. The updated four-sample XRD/XPS controls show that the Ce-containing synthesis is associated with modest lattice and surface-state changes, whereas PVP alone does not generate a resolvable new crystalline phase and is most directly supported as a morphology-regulating additive. These comparisons strengthen the separation of the two synthesis effects, but neither the Ce response nor the O 1s fitting is used to claim a quantitatively determined oxygen-vacancy concentration or proven substitutional Ce occupancy. The PVP contribution is likewise described as morphology regulation rather than a directly verified adsorption or bridging mechanism.

The optimized MnCo_2_O_4_-9 h-3%Ce-PVP electrode delivers 2008 F g^−1^ at 1 A g^−1^ and 1227 F g^−1^ at 20 A g^−1^, with 99.0% retention after 10,000 cycles at 5 A g^−1^. Kinetic analysis indicates mixed diffusion-influenced and surface-controlled charge storage, with the capacitive-controlled contribution increasing at higher scan rates. EIS supports reduced interfacial resistance and favorable apparent transport kinetics, but no independent intrinsic-conductivity value is claimed.

When paired with the carbon-supported iron oxide negative electrode, the charge-balanced asymmetric device operates over 0-1.6 V and delivers 34.6 F g^−1^ at 1 A g^−1^. It provides 12.30 Wh kg^−1^ at 0.8 kW kg^−1^ and maintains 10.60 Wh kg^−1^ at 8.0 kW kg^−1^. The 86.1% capacitance retention from 1 to 10 A g^−1^ demonstrates favorable rate capability. Although the maximum device-level energy density is moderate relative to several recent oxide-composite systems, the results show stable energy output over a broad power range together with high positive-electrode capacitance and cycling durability. All device gravimetric metrics are calculated using the total active mass of both electrodes. The negative electrode is intentionally described as carbon-supported iron oxide because its precise phase was not independently confirmed.

## Figures and Tables

**Table 1:** Device-level comparison with the recent MnCo_2_O_4_-containing and closely related asymmetric supercapacitors plotted in Figure 13e. NR indicates not reported or not directly comparable. * For BaMoO_4_/Zn-BaMoO_4_, the plotted energy/power point is calculated from the reported capacitance, voltage, and current density.

System	Voltage/Mass Basis	Energy/Power Density	Cycling	Interpretation
MnCo_2_O_4_/OMEP//AC [7]	Aqueous ASC; reported basis	11.6 Wh kg^−1^ at 8.33 kW kg^−1^	90.2%/10,000 cycles	Direct high-power MnCo_2_O_4_ benchmark
MXene/MnCo_2_O_4_//AC [29]	Aqueous ASC; reported basis	26.8 Wh kg^−1^ at 2.88 kW kg^−1^	93.8%/5000 cycles	Conductive MnCo_2_O_4_ composite
NiCo_2_O_4_/CGM//N-CGM [30]	0–1.6 V; reported basis	24.7 Wh kg^−1^ at 0.800 kW kg^−1^	85%/50,000 cycles	Related spinel-oxide/graphene ASC
MnCo_2_O_4_@NiCo-LDH//AC [31]	0–1.6 V; quasi-solid ASC	21.3 Wh kg^−1^ at 0.160 kW kg^−1^	78.7%/5000 cycles	Hierarchical MnCo_2_O_4_ heterostructure
MnCo_2_O_4_-discs//AC [32]	Aqueous ASC; reported basis	35.8 Wh kg^−1^ at 0.928 kW kg^−1^	5000 cycles; retention NR	Recent morphology-controlled MnCo_2_O_4_
MnCo_2_O_4_ nanoplatelets//AC [10]	Aqueous ASC; reported basis	34.10 Wh kg^−1^ at 0.883 kW kg^−1^	NR	Recent MnCo_2_O_4_ nanoplatelet system
BaMoO_4_/Zn-BaMoO_4_ [33]	0–1.8 V; reported basis	34.2 Wh kg^−1^ at 0.297 kW kg^−1^ *	87%/2000 cycles	Related high-voltage oxide-pair benchmark
This work	0–1.6 V; total mass of both electrodes	12.30 Wh kg^−1^ at 0.8 kW kg^−1^; 10.60 Wh kg^−1^ at 8.0 kW kg^−1^	86.1% rate retention; positive electrode: 99.0%/10,000 cycles	High-power energy retention; transparent normalization

## Data Availability

The data presented in this study are available on request from the corresponding author.
